# Activation of Granulocytes in Response to a High Protein Diet Leads to the Formation of Necrotic Lesions in the Liver

**DOI:** 10.3390/metabo13020153

**Published:** 2023-01-19

**Authors:** Ante Benić, Sanja Mikašinović, Felix M. Wensveen, Bojan Polić

**Affiliations:** Department of Histology and Embryology, Faculty of Medicine, University of Rijeka, 51000 Rijeka, Croatia

**Keywords:** high-protein diet, liver, hepatitis, inflammation, neutrophils

## Abstract

In their aspiration to become healthy, people are known to follow extreme diets. However, the acute impact on organs regulating systemic metabolism is not well characterized. Here, we investigated the acute impact of six extreme diets on the liver in mice. Most diets did not lead to clear pathology after short-term feeding. However, two weeks of feeding with a high protein diet (HPD) resulted in an acute increase of liver enzymes in the blood, indicative of liver damage. Histology revealed the formation of necrotic lesions in this organ which persisted for several weeks. Flow cytometric analysis of hepatic immune cell populations showed that HPD feeding induced activation of macrophages and neutrophils. Neutralization of the pro-inflammatory cytokine IL-1β or depletion of macrophages with clodronate-loaded liposomes or with genetic models did not ameliorate liver necrosis. In contrast, the depletion of neutrophils prevented HPD-induced hepatic inflammation. After prolonged feeding, HPD-feeding was associated with a strong increase of the cytokines IL-10 and IL-27, suggesting that anti-inflammatory mediators are activated to prevent nutrient-overload-induced damage to the liver. In summary, whereas our data indicates that most extreme diets do not have a major impact on the liver within two weeks, diets with a very high protein content may lead to severe, acute hepatic damage and should therefore be avoided.

## 1. Introduction

Diet impacts our overall well-being. It is well known that a healthy, balanced diet plays an important role in the prevention of chronic and acute diseases [1]. Unfortunately, the last few decades were marked by an alarming increase in people consuming diets that are considered unhealthy. In particular, the intake of food rich in both fat and sugar, also referred to as a ‘Western-style diet’, has led to a pandemic of obesity, metabolic syndrome, and associated diseases such as cancer [2,3]. To counter obesity, more and more people are following various fad diets that limit certain types of nutrients or restrict themselves to certain food groups only. Examples are the so-called ‘Atkin’s’ diet or the ‘keto’ diet in which people strongly restrict their carbohydrate intake whilst eating an abundance of fats and/or proteins [4]. Bodybuilders tend to favor diets with a high protein content that supposedly promote weight loss and gain of muscle mass [5]. Another modern trend are online challenges spread on social networks in which viewers are encouraged to eat unusually large quantities of specific foods (so-called ‘mukbang’ challenges). Whether these diets are efficient and safe is, however, not sufficiently investigated and their impact may be long-term [6].

Previous studies have shown [7,8,9,10] that feeding animals with diets that have an unbalanced nutrient composition can have a big impact on various aspects of body physiology. A high-fat diet (HFD) induced sterile inflammation in adipose tissue with accumulation and activation of macrophages and NK cells [9,10], ultimately leading to obesity and insulin resistance. A high cholesterol diet induced liver steatosis, insulin resistance, and aberrations in pancreatic glucose sensing [8]. The impact of ketogenic diet is much less clear. Feeding animals with a short-term (one week) ketogenic diet rich in fats and proteins, but lacking carbohydrates was reported to improve glycemic control and adipose tissue metabolism *via* its impact on resident γδ T cells [7]. Long-term feeding, on the other hand, was shown to increase systemic markers of dyslipidemia and inflammation and had a negative impact on glycemic control [11]. Others reported improved longevity and an increased health span [12]. The effects of a diet with high carbohydrate contents are also highly variable and differ with the nutrient source that is being used in excess. While 15 weeks of a high sucrose diet improved energy expenditure and insulin sensitivity without causing changes in body weight [13,14], 8 weeks of high fructose diet increased diabetes progression and caused a short-term increase in body weight [15]. A diet that is high in sodium was shown to cause an increase in circulating inflammatory CCR2^+^ Ly6C^hi^ monocytes and CD11b^+^ F4/80^+^ macrophages after 10 weeks [16], which was associated with higher levels of pro-inflammatory cytokines in circulation. At the same time, 5 weeks of a high sodium diet led to an enhanced T-cell response against tumors [17]. Clearly, more knowledge is required on the impact of diets on systemic physiology.

Insufficient attention has been given to the short-term impact of specific nutrient-rich diets on liver physiology in the first weeks after initiation. This is of particular importance as the liver serves as a hub for various metabolic processes. It has an important role in energy homeostasis via its role in glucose and fat utilization and homeostasis [18], serves as a main site of amino-acid metabolism [19], as well as having a role in the detoxification of various toxins and metabolites and an emerging immune function [20,21]. Therefore, we tested the short-term (2 weeks) effects of different extreme diets on liver physiology using a mouse model. We included diets rich in fat, cholesterol, sodium, protein, or carbohydrates to cover various nutrients which people typically eat in excess. We observed that most diets had no or only a mild impact on liver physiology over a two-week feeding period. In contrast, high-protein diet caused inflammation and acute liver pathology, which was mediated by Ly6G^+^ granulocytes. Prolonged feeding, however, caused a resolution of inflammation, which was associated with the induction of anti-inflammatory cytokines IL-10 and IL-27, most likely due to metabolic adaptation of the liver. Our findings indicate that a high protein diet may lead to acute pathological changes in the liver associated with inflammation and granulocyte activation and should, therefore, be avoided.

## 2. Materials and Methods

### 2.1. Mice

Wild-type C57BL/6J (JAX #664) mice were purchased from Jackson Laboratory (Bar Harbor, ME, USA). All genetically modified animal models were generated on the C57BL/6J background or backcrossed at least ten times with C57BL/6J mice. Mice were bred and maintained at the animal facility of the Faculty of Medicine, University of Rijeka, under specific pathogen-free conditions in 12/12-h light/dark cycles, at 21 °C and 50% humidity. Lysoyzme^Cre^ iDTR mice were created by breeding Lysoyzme^Cre^ (Lys^Cre^, JAX # 4781) heterozygous mice with homozygous Rosa iDTR/iDTR mice (JAX #7900). Animals were genotyped and both Lys^Cre^ positive animals and littermates were kept in the same cage. All animal experiments were carried out with approval from the University of Rijeka Medical Faculty Ethics Committee and Croatian Ministry of Agriculture, Veterinary and Food Safety Directorate, under number UP/I-322-01/21-01/31.

### 2.2. Diets and Feeding

The caloric value of diet was calculated using Atwater values (kcal/g) for total dry food components. All diets were autoclaved before being given to mice. Mice received food and water *ad libitum*. Mice and food were weighed twice per week.

### 2.3. In Vivo Experiments

For anti-IL-1β treatment, mice received 200 μg/mouse i.p., every three days for two weeks for the duration of feeding. For anti-Gr-1 experiment, mice received 100 µg/mouse i.p. on days 0 and 10. For elimination of macrophages using iDTR system, mice received 750 ng/mouse of diphtheria toxin i.p. on days 2, 0, and 7. For macrophage depletion, mice were injected once every two weeks with 45 mg/kg clodronate-loaded or unloaded control-liposomes (ClodronateLiposomes.com, accessed on 31 March 2022).

### 2.4. Cell Isolation and Flow Cytometry

Mice were sacrificed, serum was collected for cytokines determination, and ALT/AST assay. Alanine aminotransferase (ALT) and aspartate aminotransferase (AST) levels were determined in the Clinical Department for Laboratory Diagnostics at the Clinical Hospital Rijeka using a Cobas c 702 (Roche, Basel, Switzerland). Liver and fat pads (VAT) were isolated and weighted. Liver was homogenized and immune cells were isolated using the Percoll gradient. Cells were stained and read on a FACSAria (BD Biosciences, Franklin Lakes, NJ, USA). Gating and analysis were performed using FlowJo (TriStar, Culver City, CA, USA).

### 2.5. Histology

Pieces of liver were stored in 4% PFA for several days, then cleared and embedded in paraffin. Samples were cut using a microtome (Leica, Wetzlar, Germany) and stained with either Sirius Red or Hematoxylin and Eosin (H&E). For H&E, deparaffined slides were stained for 8 min in hematoxylin, then washed in running water for 10 min, fixed in acidified alcohol, and washed again in running water for 5 min. Then slides were stained in eosin for 2 min followed by brief washes in running and distilled water before a dip in 96% alcohol and dehydration in ethanol and xylol. For Sirius Red, deparaffined slides were stained with hematoxylin as described, followed by 1 h stain in 0.1% picro-sirius-red solution. Slides were then washed in two changes of acidified water. Slides were dehydrated in ethanol and xylol before being mounted. For histological analysis, slides were imaged by a microscope (Olympus BX51), and the entire cut was measured and analyzed using Cell^B Soft Imaging System (Olympus, Tokyo, Japan). Quantification of collagen deposits in the liver and of immune infiltration was done using Cell^B Soft Imaging System and the FIJI (ImageJ) program. Briefly, infiltrate numbers and size were quantified per mm by using the freehand ROI tool. Collagen deposits were analyzed by deconvolution of red signal and quantification of the stained area.

### 2.6. Cytokine Quantification

For the determination of cytokine levels in serum we used the LegendPlex mouse inflammation panel that enables the quantification of 13 different cytokines in the sample using flow cytometry. Sample processing was done according to the manufacturer’s protocol. In short, serum samples and assay beads were mixed in a 96-well plate and incubated for two hours. After washing, detection antibodies were added, and the plates were again incubated for 1 h. Finally, SA-PE-labeled antibodies were added to wells without washing and incubated for 30 min. Wells were washed and the plates were read on a flow cytometer (FACSVerse, BDBiosciences). Quantification and analysis were performed using LegendPlex Data Analysis Software.

For a list of reagents used, see Supplementary Table S1.

## 3. Results

### 3.1. Short-Term Extreme Diets in Mice Do Not Cause Changes in Overall Morphological Parameters

The liver controls systemic homeostasis of a wide range of metabolites, including glucose, lipids, and amino acids [19]. In addition, chronic liver disease has been associated with an electrolyte misbalance [22]. To elucidate how extreme dieting impacts liver physiology, we fed mice food containing different nutrients in high quantities (Table 1). To determine the impact of carbohydrates, we fed animals with a diet rich in fructose (HFrD). Animals received a diet rich in animal fat (HFD) or cholesterol (HChD) to determine the impact of lipids. We also provided a diet containing all three of these metabolites (SSD) to mimic a ‘Western-style’ diet [23]. To determine the impact of electrolytes, we provided a diet rich in sodium (HSD). Finally, to measure the impact of a high-protein diet (HPD), mice were fed food containing high amounts of casein, which is a diet commonly used by bodybuilders. Animals fed with a normal chow diet (NCD) were included as controls.

**Figure 1:**
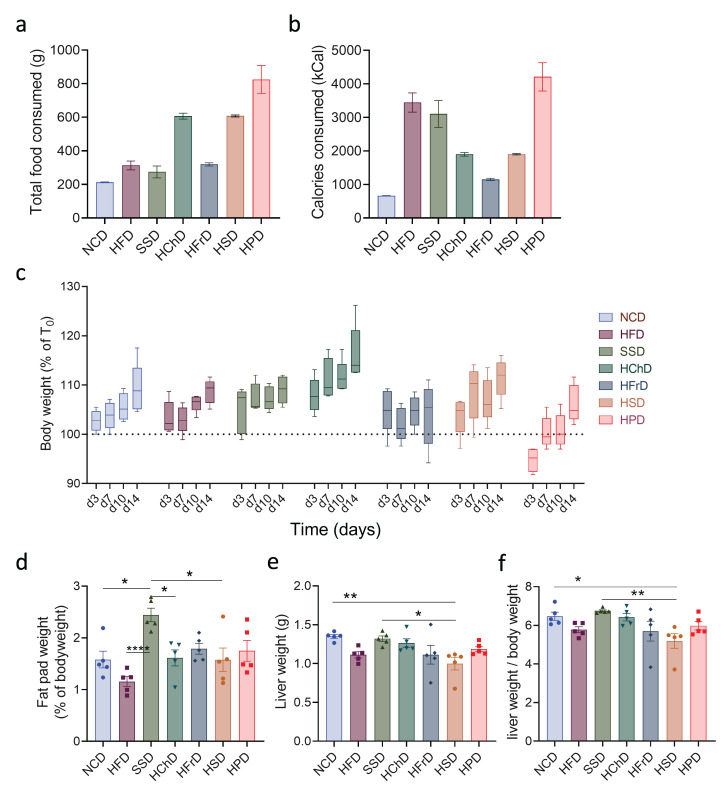
Morphological and physiological parameters of animals fed for two weeks with indicated diets. (**a**) Total food consumed and (**b**) total caloric intake during two weeks on different diets. (**c**) Change in weight of mice on different diets during the experiment. (**d**) Fat pad to body weight ratio after two weeks of diet. (**e**) Liver weight and (**f**) liver to body weight ratio after two weeks of diet. Data shown is means ± standard error of the mean (s.e.m.). For all panels, shown is one of at least two experiments with similar results. Indicated are statistical significances at ANOVA, * *p* < 0.05, ** *p* < 0.01. NCD—normal chow diet, HFD—high-fat diet, SSD—steatosis and steatohepatitis diet, HChD—high cholesterol diet, HFrD—high fructose diet, HSD—high sodium diet, HPD—high protein diet.

First, we measured the general physiological parameters of mice after two weeks of feeding with extreme diets. When we investigated the total mass of food consumed by animals, we observed large differences between the groups. Animals on HPD and HChD, in particular, were eating much more than those on a control diet (Figure 1a). Because of differences in caloric content, this also translated into large differences in the total energy consumed by animals (Figure 1b). Interestingly, we noticed that apart from small aberrations, all diets induced a similar gain in total body weight (Figure 1c). When adipose tissue weight was measured, we observed that only SSD feeding caused a significant increase compared to NCD-fed controls (Figure 1d). Thus, short-term extreme diet feeding does not directly translate into a gain in overall body weight, though it can cause increased nutrient storage in adipose tissue.

We next investigated whether extreme diets impact the liver. When analyzing total liver weight, we observed only minor changes compared to NCD-fed controls in animals eating a diet rich in sodium (Figure 1f). When liver weight was determined as a ratio of total body weight, a common parameter in humans for liver pathology [24], we again saw that only HSD-feeding caused a minor relative decrease in relative liver mass, whereas the livers from animals in all other groups were not significantly affected (Figure 1g). In summary, extreme short-term diets have a major impact on the total food consumption and caloric intake of animals. Whereas some diets can increase nutrient storage in adipose tissue, it generally does not lead to immediate changes to the overall bodyweight of animals in this time frame.

### 3.2. Short-Term High-Protein Diet Feeding Causes Necrotic Liver Lesions Associated with Immune Cell Infiltrations

**Figure 2:**
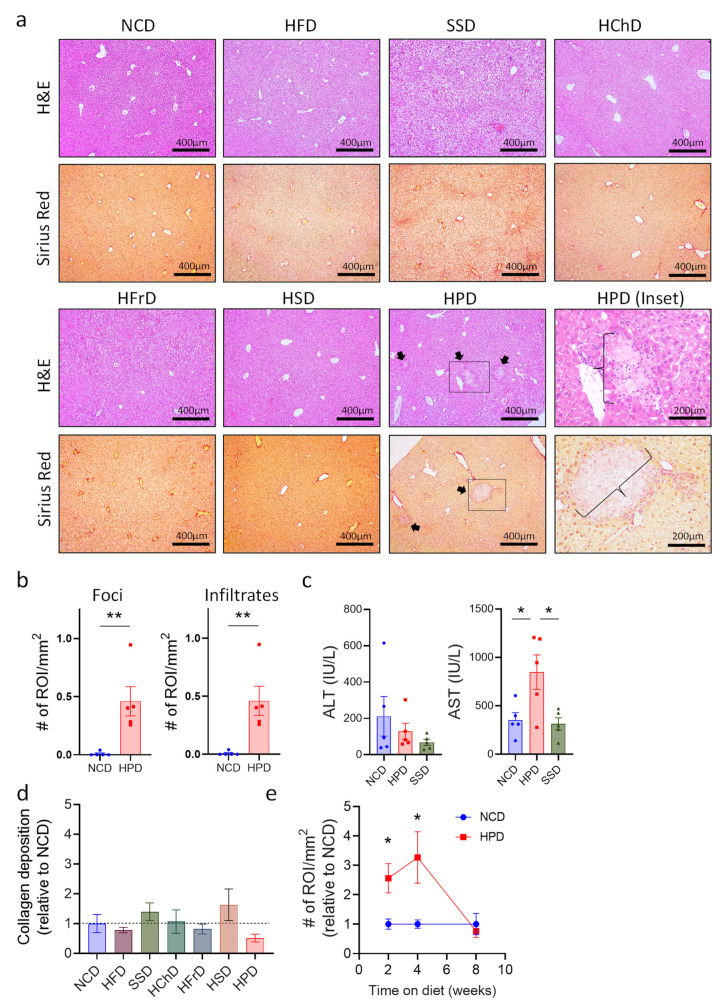
Short term high protein diet (HPD) causes changes in liver histology. (**a**) Representative H&E and Sirius Red stainings (100×) of livers after 2 weeks of seven different diets. Arrows show inflammation foci. Scale bars indicate 400 μm. For HPD-fed animals, an enlargement (200×) of necrotic areas marked by a box is shown (HPD (inset; scale bar indicates 200 μm)). Images illustrate increased immune cell infiltrates and multiple inflammatory foci (braces) in HPD samples when compared to NCD control. (**b**) Total number of foci and infiltrates per area of the slide for NCD and HPD liver sections after 2 w of feeding. (**c**) Levels of ALT and AST in serum of animals on NCD or HPD for 2 weeks. (**d**) Quantification of collagen deposits in livers of animals fed with the indicated diets. (**e**) Total number of immune cell infiltrates and inflammatory foci per liver cut area compared to NCD as baseline over time. Data shown is means ± s.e.m. For all panels, shown is one of at least two experiments with similar results. Indicated are statistical significances at ANOVA (**e**); Student’s *t*-test (**c**,**d**); * *p* < 0.05, ** *p* < 0.01.

The liver has a remarkable regenerative capacity, and even significant damage to this organ still allows it to function. Therefore, we analyzed whether extreme diets impact this organ at a microscopic level, using histology of liver sections. Histological sections were stained with H&E to show the general structure and immune cell infiltrates. Sirius red staining was used to show collagen deposits, which may lead to fibrosis if they persist for long periods. Most diets did not cause any differences in liver structure or in hepatocyte morphology (Figure 2a). Only a Western Style SSD diet and a high-protein diet caused discernable changes. In SSD liver, we observed slight ballooning of hepatocytes visible on H&E staining, which is the first stage of metabolic-associated fatty liver disease (MAFLD [25]). In contrast, livers from HPD-fed animals exhibited much more dramatic changes in liver histology, including immune cell infiltrations and large inflammation foci with hepatocyte necrosis (Figure 2a,b and Supplemental Figure S1a). Immune cell infiltrates appeared to consist mostly of myeloid cells, as determined by morphological analysis. Quantification of liver pathology showed a significant increase in both the number of necrotic lesions as well as immune cell infiltrates (Figure 2b). To confirm liver damage after two weeks of HPD feeding, we determined the levels of alanine-aminotransferase (ALT) and aspartate-aminotransferase (AST), common markers for liver injury in humans [26], in the serum of mice. The results showed a sharp increase in AST in treated animals consistent with liver inflammation, whereas the ALT levels and AST/ALT ratio were not significantly affected (Figure 2c and Supplemental Figure S1b). Animals fed with an SSD for two weeks did not show changes in liver enzymes in the serum, indicating that this diet did not cause major pathology in the investigated period (Figure 2c and Supplemental Figure S1b).

Liver pathology caused by HPD feeding was not associated with an increase of collagen deposition, as shown by Sirius red staining. Quantification of collagen deposits showed that none of the diets tested caused a significant increase compared to NCD-fed controls over a two-week time period (Figure 2a,d).

We next investigated whether prolonged HPD-feeding would aggravate liver pathology even further. Surprisingly, liver pathology decreased after 4 weeks of HPD-feeding and was mostly resolved after 8 weeks on a diet with a high protein content (Figure 2e and Supplemental Figure S1f). Body weight did not change with a prolonged diet, despite increased food and calorie intake in high protein diet-fed animals (Supplemental Figure S1d–f). Importantly, AST and ALT levels were no longer affected after 8 weeks of HPD-feeding, and also the AST/ALT ratio was not changed in these mice (Supplemental Figure S1g).

Thus, a protein-rich diet causes early-onset inflammation in the liver, which peaks at around two weeks of feeding. Upon prolonged feeding, the inflammation gets attenuated and ultimately resolved, possibly as a result of metabiotic adaptations of hepatocytes.

### 3.3. High Protein Diet Causes a Relative Increase of Pro-Inflammatory Cells of the Myeloid Lineage

**Figure 3:**
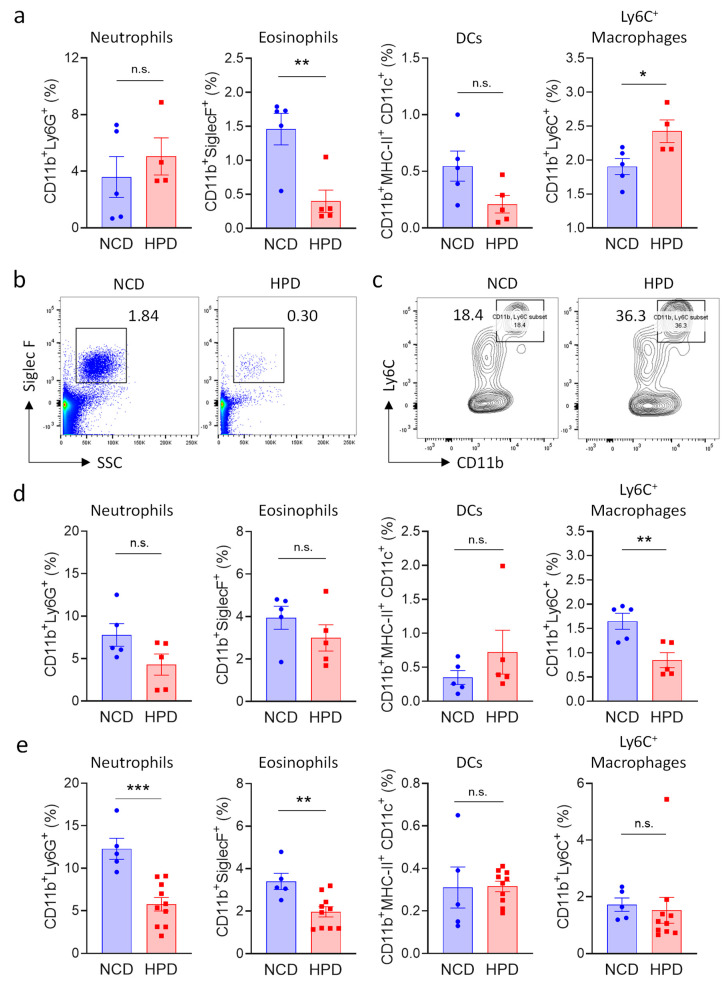
High protein diet causes transient changes in myeloid cell populations in the liver. (**a**–**c**) Frequency of myeloid subpopulations in livers of NCD or HPD-fed animals as a percentage of CD45^+^ cells. Livers were analyzed after (**a**–**c**) 2 weeks; (**d**) 4 weeks; or (**e**) 8 weeks of diet; (**a**) quantification of cell frequencies; (**d**,**e**) representative flow cytometry plot for (**b**) eosinophiles; and (**c**) Ly6C+ macrophages after 2 weeks of HPD (**b**) was gated for CD45^+^ and (**e**) for CD45^+^CD11b^+^ cells; (**d**,**e**) quantification of cell frequencies. Cells were gated according to the strategy shown in Supplemental Figure S2. Data shown is means ± s.e.m. For all panels, shown is one of at least two experiments with similar results. Indicated are statistical significances at Student’s *t*-test; * *p* < 0.05, ** *p* < 0.01, *** *p* < 0.001.

Necrotic inflammation is typically associated with an increase in myeloid immune cells number [27,28,29]; and morphological analysis revealed that these cells also accumulate in the livers of animals fed for two weeks with HPD (Figure 2a). We, therefore, investigated the impact of HPD-feeding on immune cells of the myeloid lineage. Cells were defined using established markers for neutrophils (CD11b^+^Ly6G^+^), eosinophils (CD11b^+^, SSC^High^, SiglecF^+^), dendritic cells (CD11b^+^, MHC-II^+^, CD11c^+^), and activated macrophages (CD11b^Bright^, Ly6C^+^) (Supplemental Figure S2a). Pro-inflammatory macrophages and neutrophils were increased after 2 weeks of feeding, even though the latter population did not reach statistical significance (Figure 3a–c). We observed a concomitant decrease in eosinophils, indicating that these cells are likely not involved in HPD-induced liver inflammation, thus resulting in their relative decrease. Myeloid cell populations in the spleen were not greatly affected by 2 weeks of HPD-feeding, indicating that this pathology is specifically associated with metabolic stress in hepatocytes (Supplemental Figure S2b).

To monitor changes in myeloid populations after prolonged periods of feeding, mice were kept on a HPD diet for 4 or 8 weeks. In accordance with the decrease in pathology at later timepoints, we observed that neutrophils and pro-inflammatory macrophages were progressively lost at 4 weeks. Macrophage numbers had normalized at 8 weeks, whereas neutrophil numbers were still decreased at this time point (Figure 3d,e).

In summary, short-term feeding with a high-protein diet was associated with a transient increase in pro-inflammatory cells of the myeloid lineage in the liver. Surprisingly, pro-inflammatory cells decreased at four weeks of feeding, and the hepatic immune system was mostly normalized after animals were on a HPD for 8 weeks.

### 3.4. Short-Term High-Protein Diet Is Associated with an Anti-Inflammatory Cytokine Profile

**Figure 4:**
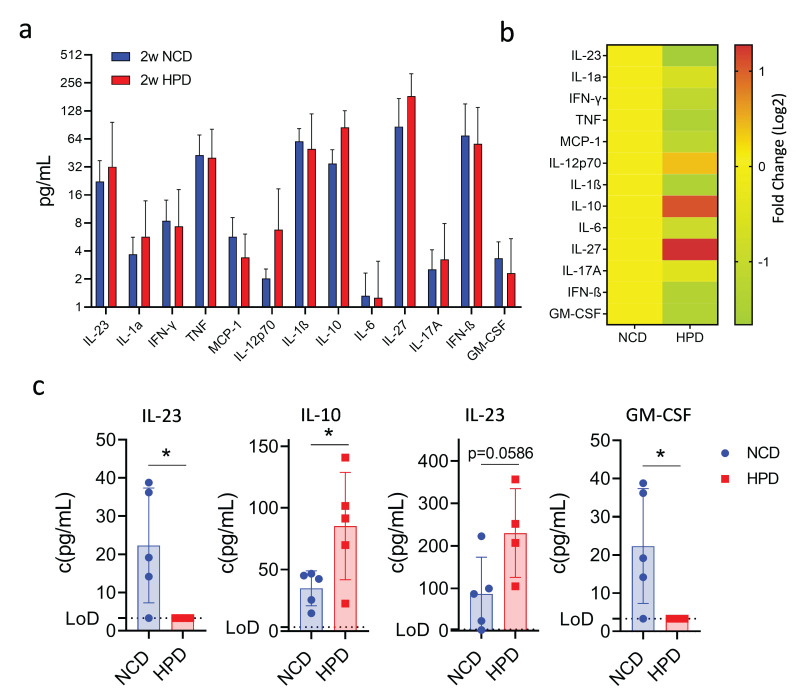
A short-term high protein diet (HPD) causes changes in the serum cytokine expression profile. (**a**) Summary of expression profiles of 13 different cytokines in serum from mice on NCD or HFD for 2 weeks. (**b**) Fold change in cytokine levels compared to NCD-fed animals. (**c**) Detailed cytokine profiles of select cytokines with differences in expression between NCD and HPD after 2 weeks of diet. IL-10, IL-27, IL-23, and GM-CSF. LoD-limit of detection. Data shown is means ± s.e.m. For all panels, shown is one of at least two experiments with similar results. Indicated are statistical significances at Student’s *t*-test; * *p* < 0.05.

Our findings show that a high-protein diet causes an initial surge of inflammation and the occurrence of necrotic lesions, which are then resolved after prolonged feeding. We, therefore, investigated the cytokine profile associated with this phenotype using a multiplex assay. Animals were fed a high-protein or normal chow diet, and cytokines were measured in the serum after 2 weeks of feeding. Out of thirteen assayed cytokines, most did not show significant changes (Figure 4a,b). Surprisingly, we did observe an increase in serum concentrations of the anti-inflammatory molecule IL-10 (Figure 4c). Levels of IL-27, a molecule well known for its ability to induce IL-10 expression [30], were also increased though it did not reach statistical significance (Figure 4c). The increase in anti-inflammatory cytokines was associated with a decrease in the pro-inflammatory cytokines IL-23 and GM-CSF, both cytokines known to promote the myeloid immune response (Figure 4c). Thus, the resolution of the inflammatory response in the liver following high-protein feeding is associated with increased production of the anti-inflammatory cytokines IL-10 and IL-27 and decreased production of the myeloid cell-stimulatory cytokines IL-23 and GM-CSF.

### 3.5. Neutrophils Cause Necrotic Liver Lesions in Response to Short-Term High-Protein Diet

**Figure 5:**
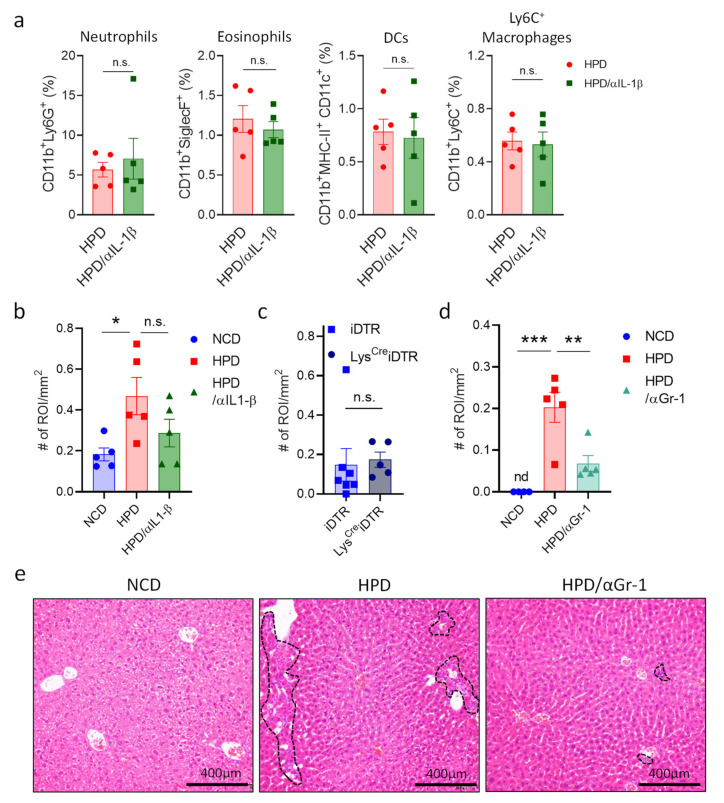
HPD-induced liver pathology is mediated by neutrophilic granulocytes and not macrophages. (**a**,**b**) Animals were fed with NCD or HPD and treated with PBS or anti-IL-1β. (**a**) Relative abundance of hepatic immune cell subpopulations; (**b**) quantification of immune cell infiltrates and inflammatory foci in livers; (**c**) Lys^Cre^iDTR and iDTR littermates were treated with diphtheria toxin and fed with HPD. After 2 weeks, immune cell infiltrates and inflammatory foci in livers were quantified; (**d**,**e**) animals were fed with NCD or HPD and treated with PBS or anti-GR-1; (**d**) quantification of immune cell infiltrates and inflammatory foci in livers; (**e**) representative images of H&E stained liver slides. Dashed lines indicate sites of immune cell infiltration. Scale bars indicate 400 μm. All cells were gated according to the strategy shown in Supplemental Figure S2. Data shown is means ± s.e.m. For all panels, shown is one of at least two experiments with similar results. Indicated are statistical significances at Student’s *t*-test; * *p* < 0.05, ** *p* < 0.01, *** *p* < 0.001.

Having discovered the cytokines responsible for resolving necrotic liver inflammation, we next wanted to investigate which cells are responsible for the hepatic damage in response to a short-term high-protein diet. IL-1β is one of the main pro-inflammatory signals in liver inflammation that plays a key detrimental role in multiple conditions [31]. To determine if this cytokine mediates HPD-induced liver pathology, mice were put on an HPD diet for two weeks with or without treatment with an antibody neutralizing IL-1β. After two weeks of HPD, we analyzed immune cell abundance in the liver, as well as liver histology. There were no significant differences in the relative abundances of different myeloid cell subsets in HPD-fed mice treated with PBS or an IL-1β neutralizing antibody (Figure 5a). However, there did seem to be a slight improvement in liver pathology with a reduction in inflammation and cell infiltration, even though this did not reach statistical significance (Figure 5a,b and Supplemental Figure S3a,b).

In addition to IL-1β, macrophages mediate inflammation through a multitude of other factors. We, therefore, decided to eliminate these cells to investigate whether they are important for mediating HPD-induced pathology. To deplete macrophages, we made use of two different strategies. First, we injected mice with control liposomes or liposomes filled with clodronate to deplete macrophages and fed them with an HPD for two weeks. Next, livers were analyzed by histology. No differences were observed between the two groups (Supplemental Figure S3c). Next, we made use of the Lys^Cre^iDTR model. In this system, macrophages can be eliminated upon the administration of diphtheria toxin. Lys^Cre^iDTR mice and iDTR littermates were injected with diphtheria toxin and fed with an HPD for two weeks. After two weeks, livers were analyzed by histology. However, histological analysis showed no change in the number of infiltrates between the two groups (Figure 5c). Thus, whereas pro-inflammatory macrophages increase in the liver following HPD-feeding, they do not appear to be directly responsible for the induction of pathology.

Neutrophils can induce liver damage and promote hepatic inflammation in the context of viral infection [27]. We, therefore, investigated the role of neutrophils in HPD-induced liver inflammation. Animals were treated every other day with a depleting antibody directed against GR1 (Ly6G) and a few with an HPD for two weeks. Next, liver pathology was analyzed by histology. This revealed that neutrophil depletion resulted in a significant reduction in liver inflammation when compared to HPD-fed mice without depleting antibodies (Figure 5d,e and Supplemental Figure S3d), with a strongly decreased number of immune cell infiltrates.

Thus, short-term high-protein feeding causes acute necrotic liver inflammation which is independent of macrophages and IL-1β but depends on the action of neutrophilic granulocytes. With continued feeding, however, the liver adapts to a higher protein load [32], which is associated with a resolving of inflammation, possibly through the concerted action of IL-27 and IL-10.

## 4. Discussion

Extreme diets are an increasingly common habit in our society but their impact on general health has been incompletely investigated. Here we studied the effects of specific nutrient overload on the liver. We find that most nutrients do not have a discernable impact on the liver after a 2-week feeding regiment. The combined overload of cholesterol, fructose, and fat—a ‘Western-style diet’—did cause hepatocyte ballooning, but the effects were very mild at this time point. In contrast, a high protein diet caused pathology in the liver, characterized by immune cell infiltration and the occurrence of necrotic and inflammatory lesions. Surprisingly, the liver was able to adapt, and inflammation was resolved over time. Our findings illustrate the remarkable adaptive capacity of liver metabolism but indicate that extreme diets are not without a risk to the immediate health of people.

The impact of high-protein diets on general wellbeing is still a matter of debate. Some studies have shown it to be beneficial as it was able to reduce adiposity and weight gain induced by a high-fat diet [33]. Other diets, such as a high sucrose diet did not have this positive effect [34,35,36]. The effects of HPD appear to differ between organ systems, as this diet was shown to induce gut [37] and systemic [38] inflammation with alterations in microbiota after long-term feeding. In humans with non-alcoholic fatty liver disease (NAFLD), a high-protein diet was shown to correlate with higher disease scores [39]. Concordant with our results showing an increase in liver inflammation, four months of high protein diet induced triglyceride deposition and an increase of markers of inflammation and liver injury such as AST, CRP, TNFα, and HSP90 in serum [40]. Thus, whereas HPD may correct some negative effects of nutrient overload possibly by reprogramming hepatic metabolism, it is associated with an overall pro-inflammatory profile.

An unanswered question is how protein overload leads to necrotic inflammation in the liver. One explanation may be an increase in cellular stress due to an overload of amino-acid catabolic metabolism. Unlike fats or sugars, amino acids are not stored in the body. Rather, their level is kept steady by the balance of catabolic and anabolic processes, most of which take place in the liver [41]. When confronted with an excess of protein, the liver increases the rate of degradation and switches to a different energy metabolism [32,42]. Most notably, the expression of proteins involved in nitrogen processing is induced, causing an increase in ammonia and urea levels in the liver and blood [40,43]. In several different studies, aberrations in the urea cycle were connected with higher rates of liver inflammation, increased liver disease burden, and liver pathologies [44,45,46,47]. This mechanism could explain the observed increase in inflammation upon the start of a high-protein diet via the activation of innate immune cells. It is known that pro-inflammatory Gr-1^hi^ monocytes are recruited into the liver upon acute liver injury [29]. Recruitment of neutrophils worsens liver injury in an antigen-non-specific manner during viral hepatitis, and they are an important source of pro-inflammatory cytokines [27,28]. This would explain the observed amelioration of liver infiltration upon blocking of Gr-1 in our experiments.

Surprisingly, HPD-induced liver inflammation was transient and resolved itself within 8 weeks of feeding. When analyzing the cytokine profile, we observed an increase in IL-10 and IL-27, whereas IL-23 was decreased. IL-23 is a pro-inflammatory cytokine linked to IL-12 and is produced by activated myeloid cells, including macrophages. IL-23 has been known to be produced as a response to damaged hepatocytes and induces both T_H_1 and T_H_17 responses, including an increase in apoptosis and necrosis [48,49,50]. The reduced levels of this cytokine at two weeks of feeding, along with the observed amelioration of liver pathologies in later time points, would indicate that IL-23 performs its role at early stages of the inflammatory response but is subdued by anti-inflammatory mechanisms at later time points. We propose that these anti-inflammatory mechanisms involve IL-10 and IL-27. IL-10 is known to be anti-inflammatory and anti-fibrotic in the liver [51] and was shown to induce senescence in activated hepatic stellate cells in response to CCl_4_-induced liver injury [52]. Similarly, IL-27 is produced by macrophages and has an anti-viral and anti-inflammatory effect [53]. It has also been shown to promote liver regeneration in tandem with IL-17 in the choline-deficient, ethionine-supplemented model of liver injury [54]. Therefore, the observed increase of these two anti-inflammatory cytokines, along with the decrease in IL-23, would indicate the start of the resolution of inflammation in the liver caused by a high-protein diet.

An important question is whether our observations can be translated to the human situation. Indeed, a 14-day high-casein diet was shown to increase hepatic nitrogen clearance in people, which was impaired in people with cirrhosis, indicating the ability and necessity of the liver to adapt to higher protein intake [55]. In addition, changes in the urea cycle are associated with increased liver enzymes in serum and fibrosis in patients with non-alcoholic fatty liver disease [56]. Importantly, the disease score in patients with NAFLD, as determined by biopsy, was worse in patients with a high daily relative intake of proteins [39]. Thus, urea appears to have a negative impact on the liver, especially in the context of pre-existing pathology. However, whether a short-term high-protein diet causes transient liver pathology in humans remains to be investigated.

## 5. Conclusions

Our findings indicate that a high protein diet induces acute liver damage at the onset of feeding, possibly by metabolic stress due to an overload of amino acid metabolism in the liver. This metabolic stress initiates inflammation mediated by neutrophilic granulocytes. As the liver adapts to the new metabolic state, inflammation is resolved, which is associated with an increase in anti-inflammatory cytokines (Supplementary Figure S4). Our findings indicate that diets with a high protein content can have a significant detrimental impact on liver physiology and should, therefore, not be consumed by people with pre-existing hepatic pathology.

## Figures and Tables

**Table 1:** Diet composition.

Diet Name	Diet Composition	(kcal/g)
Normal chow diet (NCD)	Mucedola 4RF21 diet 18.5% *w*/*w* crude protein 3% *w*/*w* crude fats and oils 6% *w*/*w* crude fibers 7% *w*/*w* crude ash 53% *w*/*w* NFE (carbs): −42.6% starch −3.7% sucrose	3.13
High fat diet (HFD)	60% *w*/*w* NCD pellets 40% *w*/*w* fat (lard)	11
Steatotic diet (SSD)	40% *w*/*w* NCD pellets 40% *w*/*w* fat (lard) 20% *w*/*w* fructose 2% *w*/*w* cholesterol	11.3
High cholesterol diet (HChD)	NCD pellets 2% *w*/*w* cholesterol	3.13
High fructose diet (HFrD)	40% NCD pellets 60% fructose	3.6
High sodium diet (HSD)	NCD pellets 4% NaCl 1% NaCl in drinking water	3.13
High protein diet (HPD)	50% *w*/*w* NCD pellets 50% *w*/*w* casein powder	5.1

## Data Availability

The data presented in this study are available in article and supplementary materials. Further information and requests for resources and reagents should be directed to and will be fulfilled by the lead contact, Bojan Polić (bojan.polic@uniri.hr).

## Supplementary Materials

The following supporting information can be downloaded at: https://www.mdpi.com/article/10.3390/metabo13020153/s1, Figure S1: Short term high protein diet (HPD) causes changes in liver histology; Figure S2: (a) Gating strategy for analysis of myeloid cell populations in the liver. (b) Relative frequency of indicated immune cell subpopulations in the spleen of NCD or HFD-fed animals after 2 weeks of diet; Figure S3: HPD-induced liver pathology is mediated by neutrophilic granulocytes; Figure S4: Graphical representation of transient HPD-induced liver damage. Table S1: List of reagents and resources.
