# Supplementary material for: Activation of Granulocytes in Response to a High Protein Diet Leads to the Formation of Necrotic Lesions in the Liver
Source: Metabolites. 2023 Jan 19;13(2):153. doi: 10.3390/metabo13020153 (PMC9962952; doi:10.3390/metabo13020153)
Supplement: Supplementary file 1 [file metabolites-13-00153-s001.zip › metabolites-2149091-supplementary.pdf]

Article

Activation of granulocytes in response to a high protein diet leads to the formation of necrotic lesions in the liver

Ante Benić[1], Sanja Mikašinović, Felix M Wensveen[1], Bojan Polić[1,*]

Supplemental Material.

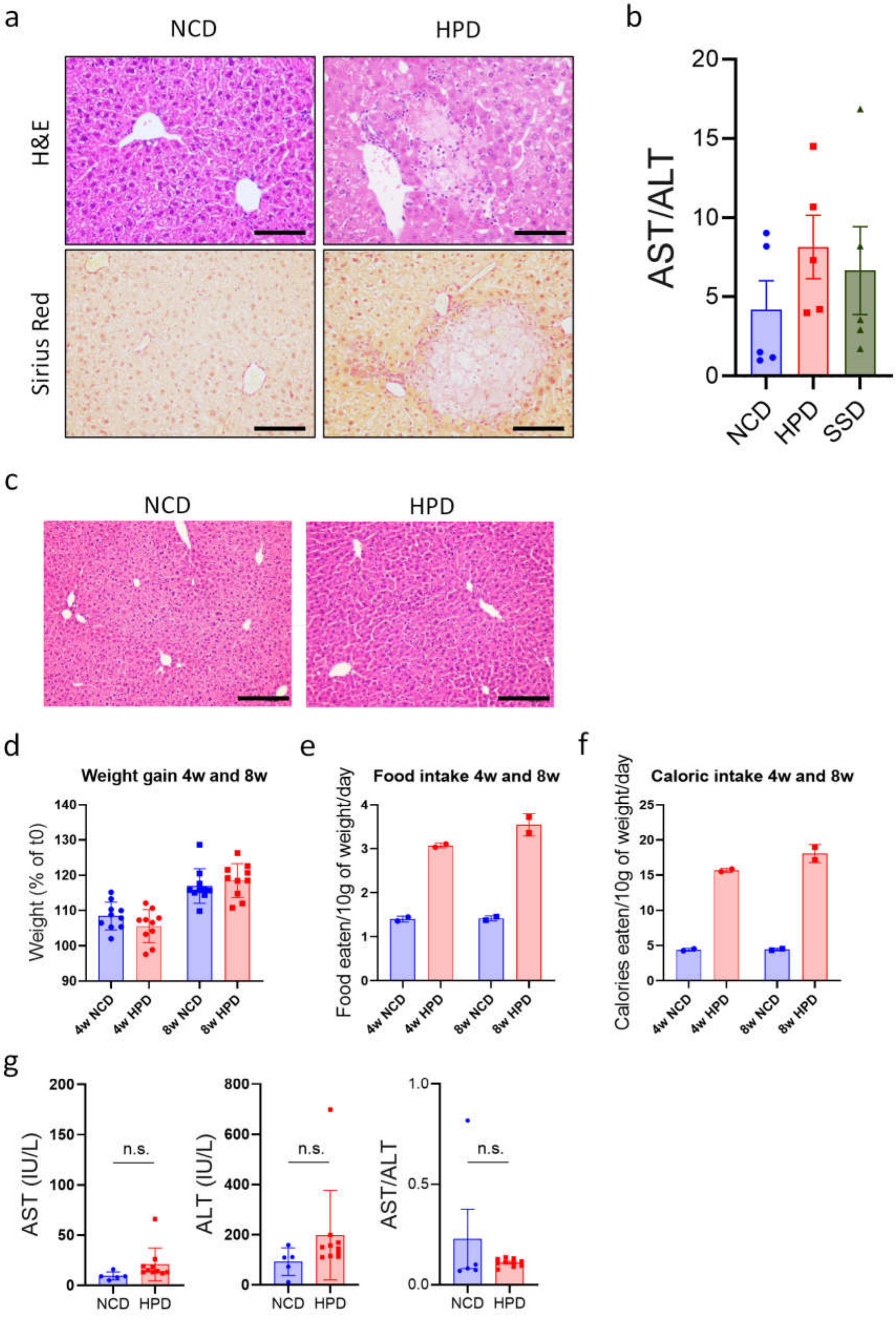
a
NCD
HPD
H&E
Sirius Red
b
AST/ALT
20
15
10
5
0
NCD
HPD
SSD
c
NCD
HPD
d
Weight gain 4w and 8w
Weight (% of t0)
140
130
120
110
100
90
4w NCD
4w HPD
8w NCD
8w HPD
e
Food intake 4w and 8w
Food eaten/10g of weight/day
4
3
2
1
0
4w NCD
4w HPD
8w NCD
8w HPD
f
Caloric intake 4w and 8w
Calories eaten/10g of weight/day
25
20
15
10
5
0
4w NCD
4w HPD
8w NCD
8w HPD
g
AST (IU/L)
200
150
100
50
0
n.s.
NCD
HPD
ALT (IU/L)
800
600
400
200
0
n.s.
NCD
HPD
AST/ALT
1.0
0.5
0.0
n.s.
NCD
HPD

Supplemental figure S1. Short term high protein diet (HPD) causes changes in liver histology. (a) Enlargement of livers stained with H&E or Sirius Red after 2 weeks of NCD or HPD. Scale bars indicate 200 µm. (b) AST/ALT in the serum of animals fed for 2 weeks with an NCD, HPD or SSD. (b) AST/ALT ration in serum of animals fed for 2 weeks with indicated diets. (d) Weight gain compared to starting weight in animals fed NCD or HPD for 4 and 8 weeks. (e) Food intake and (f) Atwater caloric intake at 4 weeks or 8 weeks of NCD and HPD. (f) Representative slide of livers stained with H&E after 8 weeks of NCD or HPD. Scale bars indicate 400 µm. (g) AST and ALT in serum of animals fed for 8 weeks with an NCD or HPD. Data show means ±s.e.m. For all panels, shown is one of at least two experiments with similar results.

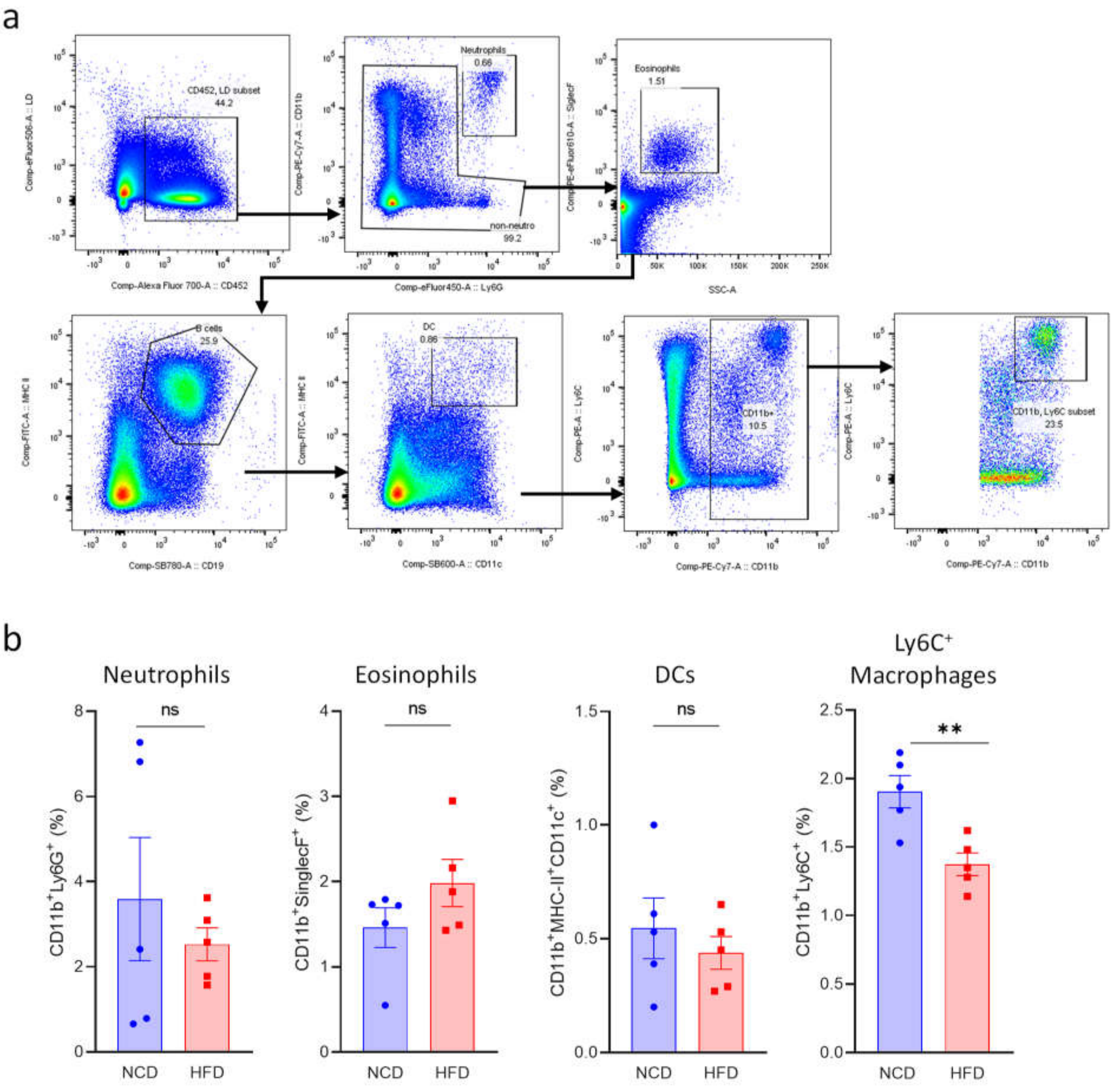

Supplemental figure s2. (a) Gating strategy for analysis of myeloid cell populations in the liver. (b) Relative frequency of indicated immune cell subpopulations in the spleen of NCD or HFD-fed animals after 2 weeks of diet. Data show means ±s.e.m. For all panels, shown is one of at least two experiments with similar results. Indicated are statistical significances at Student's t-test; * p<0.05, ** p<0.01, *** p<0.001.

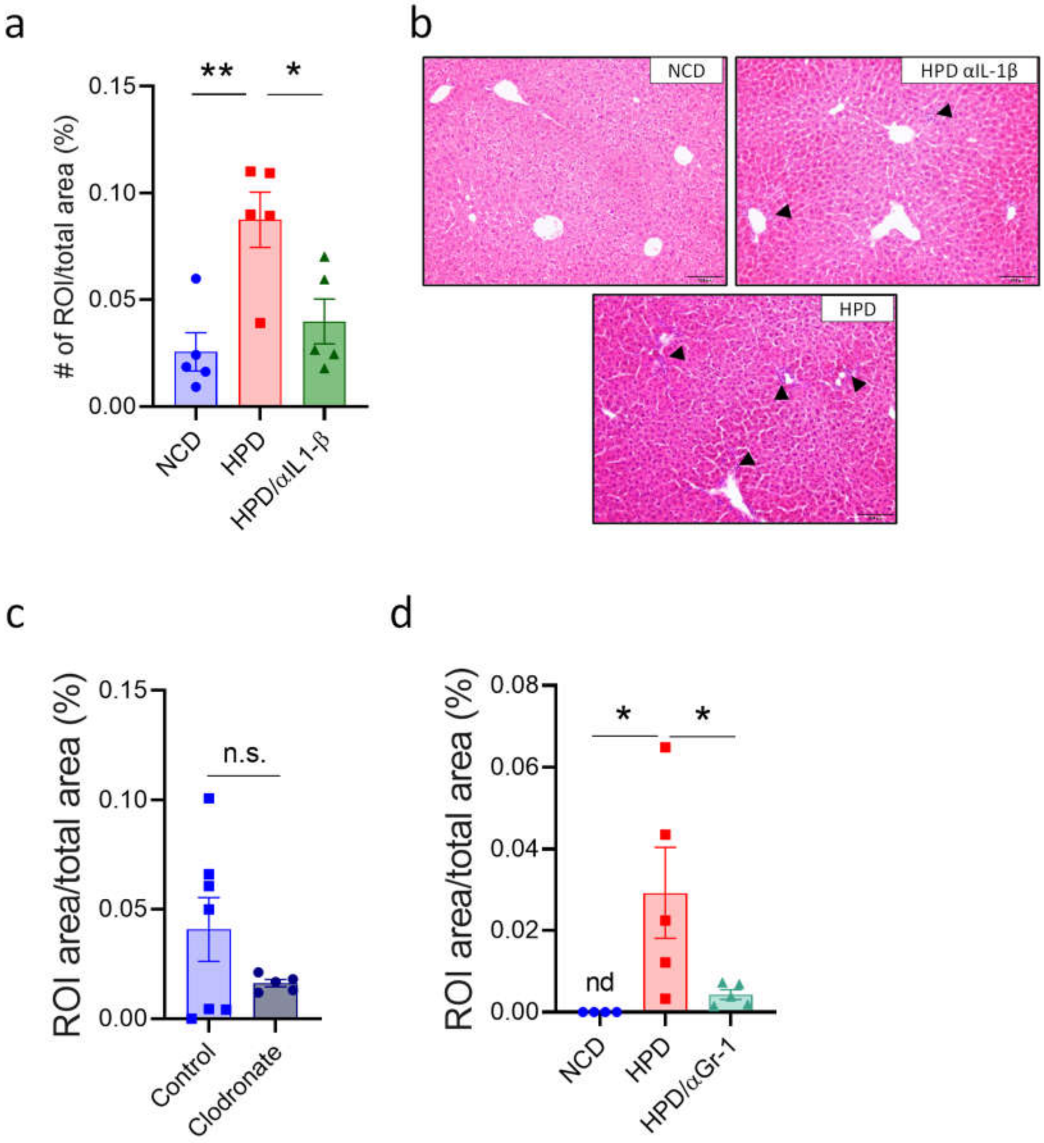

Supplemental figure s3. HPD-induced liver pathology is mediated by neutrophilic granulocytes. (a-b) Animals were fed for 2 weeks with an NCD or HPD and treated with PBS or anti-IL-1β. (a) Immune infiltration was calculated as a percentage of the total area of the section. (b) Representative liver slides stained with H&E. (c) WT mice were injected once every three days with empty (control) or clodronate-filled liposomes to deplete macrophages and feed a HPD an livers were analyzed by histology after two weeks. Immune infiltration was calculated as a percentage of the total area of the section (d) Animals were fed for 2 weeks with an NCD or HPD and treated with PBS or anti-GR-1. Immune infiltration was calculated as a percentage of the total area of the section. Data show means ±s.e.m. For all panels, shown is one of at least two experiments with similar results. Indicated are statistical significances at Student's t-test; * $p<0.05$, ** $p<0.01$, *** $p<0.001$.

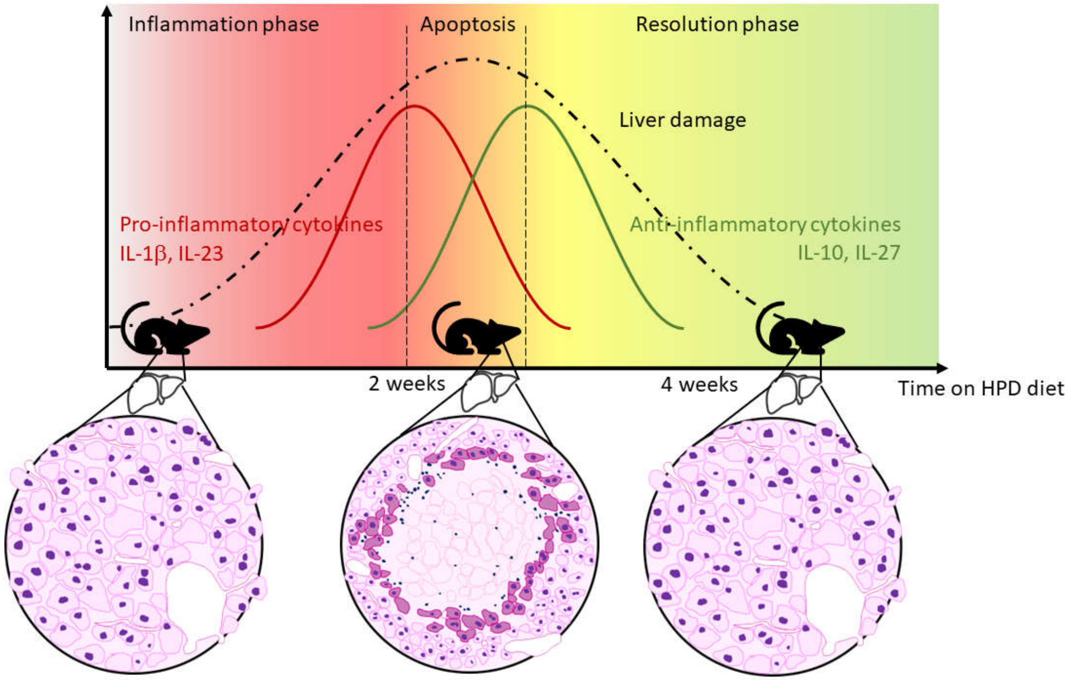

Supplemental figure s4. Graphical representation of transient HPD-induced liver damage.

Supplementary Table s1; List of reagents and resources.

REAGENT or RESOURCE	SOURCE	IDENTIFIER
**Antibodies**		
Anti-mouse IL-1β (clone B122)	BioXCell	BE0246
Anti-mouse Ly6G/Ly6C (Gr-1) (clone RB6-8C5)	BioXCell	BP0075
Armenian hamster anti-mouse CD11c monoclonal antibody (clone N418)	ebioscience	63-0114-82
Mouse anti-mouse CD45.2 monoclonal antibody (clone 104)	ebioscience	56-0454-82
Rat anti-mouse CD11b monoclonal antibody (clone M1/70)	ebioscience	25-0112-82
Rat anti-mouse CD19 monoclonal antibody (clone eBio1D3)	ebioscence	78-0193-82
Rat anti-mouse F4/80 monoclonal antibody (clone BM8)	ebioscience	17-4801-82
Rat anti-mouse Ly6C monoclonal antibody (clone HK1.4)	ebioscience	12-5932-82
Rat anti-mouse Ly6G monoclonal antibody (clone 1A8-Ly6g)	ebioscience	48-9668-82
Rat anti-mouse MHC-II (I-A) monoclonal antibody (clone NIMR-4)	ebioscience	11-5322-82
Rat anti-mouse Siglec F monoclonal antibody (1RNM44N)	ebioscience	46-1702-82
**Chemicals, Peptides, and Recombinant Proteins**		
Casein	Myprotein THG	
Cholesterol	Ssniff	R143C010
Clondronate and control liposomes	Liposoma	CP-005-005
Diphtheria toxin	Merck	322326
Entellan	Sigma Aldrich	1079600500
Eosin	Fisher Scientific	10061830
Fixable Viability Dye eFluor 506	ebioscience	65-0865-14
Formaldehyde (4% NB)	Biognost	FNB4
Fructose	Ssniff	R111F040
IC Fixation buffer	Invitrogen	00-8222
Mayer's hematoxylin	Sigma Aldrich	MDL# MFCD00078111
Percoll	Cytiva	17089101
Permeabilization Buffer (10x)	Invitrogen	00-8333
Picric acid	Sigma Aldrich	319287
Sirius Red (Direct Red 80)	Sigma Aldrich	365548
Standard mouse diet pellets	Mucedola	Diet 4RF21
SuperBright Complete Staining Buffer	ebioscience	SB-4401-75
**Critical Comercial Assays**		

Legendplex Mouse Inflammation Panel (13-plex)	BioLegend	740446
**Software and Algorithms**		
Cell^ B Soft Imaging System	Olympus	
Fiji/ImageJ	Open Source; doi:10.1038/nmeth.2019	https://imagej.net/software/fiji/
FlowJo	FlowJo LLC, BD Life Sciences	
GraphPad Prism	GraphPad Software	
**Experimental Models: Organisms/Strains**		
C57BL/6 (B6; line 664)	Jackson Laboratories	JAX: 000664
Lyz Cre	Jackson Laboratories	JAX: 0118956
Rosa26 iDTR	Prof. Ari Waisman; Mainz, Germany	JAX: 007900
